# Combination antiretroviral therapy and chronic HIV infection affect serum retinoid concentrations: longitudinal and cross-sectional assessments

**DOI:** 10.1186/1742-6405-9-3

**Published:** 2012-02-01

**Authors:** Maude Loignon, Hélène Brodeur, Sonia Deschênes, Denis Phaneuf, Pangala V Bhat, Emil Toma

**Affiliations:** 1 Research Centre, Centre Hospitalier de l'Université de Montréal (CHUM) - Hôtel-Dieu, Montréal, H2W 1T7, Canada; 2 Department of Microbiology & Infectious Diseases, CHUM- Hôtel-Dieu, Montreal, H2W 1T8, Canada; 3 Department of Medicine, University of Montreal, Montreal, H3C 3J7, Canada; 4 Department of Microbiology & Immunology, University of Montreal, Montreal, H3C 3J7, Canada

**Keywords:** Retinoic acids, Retinol, Combination antiretroviral therapy, Chronic HIV infection

## Abstract

**Background:**

Several lines of evidence suggest that retinoids (retinol-ROL or vitamin A, and its active metabolites, retinoic acids-RAs) play important pathogenic roles in HIV infection and combination antiretroviral therapy (cART)-related events. We previously reported that antiretrovirals alter RAs synthesis *in vitro*. We hypothesised that *in vivo *serum retinoid concentrations are affected by both cART and HIV infection. This might explain several clinical and laboratory abnormalities reported in HIV-infected patients receiving cART.

**Methods:**

The effects of optimal cART and chronic HIV on serum retinoids were firstly assessed longitudinally in 10 HIV-infected adults (group1 = G1): twice while on optimal cART (first, during long-term and second, during short term cART) and twice during 2 cART interruptions when HIV viral load (VL) was detectable. Retinoid concentrations during optimal long term cART in G1 were compared with cross-sectional results from 12 patients (G2) with suboptimal cART (detectable VL) and from 28 healthy adults (G3). Serum retinoids were measured by HPLC with ultraviolet detection. Retinoid concentrations were correlated with VL, CD4^+ ^T- cell count and percentages, CD8^+^38^+ ^fluorescence, triglycerides, cholesterol and C-peptide serum levels.

**Results:**

During optimal cART, G1 participants had drastically reduced RAs (0.5 ± 0.3 μg/dL; P < 0.01) but the highest ROL (82 ± 3.0 μg/dL) concentrations. During cART interruptions in these patients, RAs slightly increased whereas ROL levels diminished significantly (P < 0.05). G3 had the highest RAs levels (7.2 ± 1.1 μg/dL) and serum ROL comparable to values in North Americans. Serum ROL was decreased in G2 (37.7 ± 3.2 μg/dL; P < 0.01). No correlations were noted between RA and ROL levels or between retinoid concentrations and CD4^+ ^T- cell count, CD8^+^38^+ ^fluorescence, VL. ROL correlated with triglycerides and cholesterol in G1 (r_s _= 0.8; P = 0.01).

**Conclusions:**

Serum RAs levels are significantly diminished by cART, whereas ROL concentrations significantly decreased during uncontrolled HIV infection but augmented with optimal cART. These alterations in retinoid concentrations may affect the expression of retinoid-responsive genes involved in metabolic, hormonal and immune processes and be responsible for some adverse events observed in HIV-infected persons treated with antiretrovirals. Further studies should assess concomitant serum and intracellular retinoid levels in different clinical situations in larger, homogenous populations.

## Introduction

Retinoids (retinol-ROL- or vitamin A, and its main active metabolites, retinoic acids-RAs) play key roles in multiple human processes [1-3]. ROL is reversibly oxidized intracellularly to retinal by alcohol and short-chain dehydrogenases, whereas retinal is irreversibly oxidized by cytosolic retinal aldehyde dehydrogenases (RALDHs) to RAs, mainly all-*trans *RA and 9-cis RA [1-3]. Intracellular concentrations of RAs are tightly regulated by synthesizing and catabolizing enzymes and by their binding to cytosol RAs-binding proteins (CRABPs) [2,4]. RAs enter the nucleus, bind and activate nuclear RAs receptors (RARs) or/and retinoid X receptors (RXRs) [3-5]. All-*trans *RA is the physiological ligand for RAR, whereas 9-cis RA is a high-affinity ligand, i*n vitro*, for RXRs and for RAR [4,5]. However, *in vivo*, biological activity of 9-cis-RA is not firmly established [3-5]. Once activated by their ligands, these nuclear receptors (ligand-activated transcription factors) bind to RA response elements (RAREs) in the promoter/enhancer of a multitude of genes involved in lipid, glucose and hormonal metabolism, innate and adaptive immunity [1-6]. RXRs also form heterodimers with other nuclear receptors acting as transcription factors for other multiple genes involved in metabolic, hormonal and immune processes [4-9]. Responses in gene expression depend on intracellular RAs concentrations [4,5,9]. Alteration of retinoid concentrations could have, therefore, multiple consequences.

RAs are used therapeutically for promyelocytic leukemia, T-cell lymphoma, psoriasis, severe nodular acne, and Kaposi's sarcoma [6,10-12]. Several adverse effects have been reported with their use, such as desquamative cheilitis, xerosis, alopecia, pyogenic granuloma of nail folds, and hyperlipidemia [11,12]. Similar features, called retinoid-like adverse effects, have been observed during combination antiretroviral therapy (cART), especially when certain HIV protease inhibitors (PIs) were included in the therapeutic regimen [12-14]. These clinical manifestations are often associated with morphological and metabolic abnormalities [13-15]. It has been proposed that PIs interfere with retinoid and lipid metabolism [15], and heightened retinoid signalling has been indirectly attributed to the protease inhibitor indinavir [16].

We demonstrated that RAs synthesis is altered *in vitro *by antiretrovirals which increased RALDH1's activity and expression, the main RA-synthesising enzyme [17].

Although ROL status has been evaluated in HIV infection [18,19], no investigation of serum RAs has been undertaken thus far, in spite of their recognized implications in HIV infection and several cART-related events.

Here, we report the effects of both long-term and short term optimal cART (i.e. HIV viral load-VL- below detection limit), and of HIV during cART interruptions (when VL is detectable) on retinoid concentrations in HIV-infected adults from prospective, longitudinal assessments in the same study participants (an intra-subject approach). The effects of optimal cART on retinoids in this group of patients were compared with results in patients with suboptimal cART (repeated, detectable VL) and healthy adult volunteers. Correlations were made with immuno-virological (i.e. CD4^+ ^cell count and percentages, CD8^+^38^+ ^fluorescence index and VL) results as well as with main metabolic parameters (cholesterol, triglycerides and, in HIV-infected persons, C-peptide), which could be affected by cART or HIV. We found that both uncontrolled HIV infection and cART affect retinoid concentrations in HIV-infected adults. These changes in retinoid concentrations might explain several HIV- and cART-related clinical events, as well as some metabolic, hormonal and immune abnormalities, reported in HIV-infected individuals receiving cART.

## Methods

### Participants

Prospective, longitudinal assessments were undertaken in 10 HIV-infected participants at a Canadian HIV Trials Network (CTN) study on therapeutic vaccination and cART interruptions (group 1 = G1). This was a 5½ year proof-of-concept trial (CTN-140) conducted between 2000 and 2006 and extended to 2010 for long-term follow-up [Toma E. *et al*.: manuscript in preparation]. Its main objective was to explore whether cART exposure could be minimized by therapeutic vaccination with Remune™ initiated after targeting HIV reservoirs (by intensifying an already optimal cART), and after reducing immune activation using hydroxyurea for 5 months before the first dose of Remune™. Therapeutic vaccine was administered every 3 months for 3 years and individualized intermittent cART interruptions and cART reinitiations were performed according to predefined criteria. The primary end-point for CTN-140 was the time spent without antiretrovirals. Viro-immunological effects and clinical outcome were secondary end-points.

We have taken advantage of the design of this trial to explore longitudinally in the same patients the effects of both cART and HIV (during cART interruptions) on retinoid concentrations at 4 time points: ON 1 = during intensification period of a prolonged and optimal cART (long-term effect); OFF 1 = during a first cART interruption when VL was detectable; ON 2 = on re-initiated cART when VL was again below detection limit (short-term effect); OFF 2 = during a second cART interruption when VL was again detectable.

Serum retinoids concentrations in G1 during cART intensification (ON 1) were compared with those in 12 HIV-infected patients with suboptimal cART having repeated detectable VL, followed at the same outpatient clinic (G2) and 28 healthy adult volunteers (G3). To reduce selection bias, patient recruitment for G2 and healthy volunteers for G3 was undertaken in consecutive order (either when they came to the clinic or as they agreed to participate, respectively) in the months preceding serum retinoid assessments. Since this was an exploratory work, we did not match the participants by gender or by age. However, a separate analysis was performed for males only (10 in each group) and between healthy males and females volunteers.

This study was conducted according to the guidelines laid down in the Declaration of Helsinki and all procedures involving patients and healthy volunteers, the protocol, consent forms and amendments were approved by the Research Ethics Committee of the Centre Hospitalier de l'Université de Montréal (CHUM). All participants provided written, informed consent.

### Serum retinoid levels

Blood was collected after overnight fasting in vacutainer tubes containing a silica gel-based clotting activator and previously wrapped with aluminium foil to minimize light exposure. The samples were processed in a dark room, and serum was obtained by centrifugation at 2 620 g at 4^°^C, and then stored in 1.5 ml brown Eppendorf cryotubes at -80^°^C until assayed. Retinoids from the serum samples were extracted by butanol/acetonitrile (equal volumes) essentially as described by McClean et al. [20] except that the method was applied for smaller sample volumes [21-23]. At the time of assay, samples stored at -80^°^C were defrosted on ice and centrifuged for 10 min at 4^°^C at 2 620 g to obtain clear supernatants. 200 μl of serum was transferred to a borosilicate tube wrapped in aluminium foil, and 200 μl of butanol/acetonitrile (1:1) was added. The mixture was vortexed for 1 min and vortexed again for 30 s. The extraction mixture was centrifuged at 2 620 g for 15 min in a Sorval RC3C Plus centrifuge pre-cooled to 4^°^C. 100 μl of clear supernatant was injected into the HPLC system. Recovery studies were performed with the addition of retinoids (5-50 ng/100 μl range) to 3 separate serum samples. Retinoid recovery in this extraction method was approximately 99% [21-23].

The HPLC system consists of a Shimadzu Model LC-10ADVP (Mandel, Guelph, ON, Canada) equipped with a SIL-HTC autosampler and cooling system [22-24]. Retinoids were separated on a Phenomenex 10-ODS analytical column (250 × 4.5 mm, Phenomenex, Inc., Torrance, CA), and eluted with a mobile phase of acetonitrile: water (60:40) containing 10 mM ammonium acetate at a flow rate of 1.2 mL/minute. Retinoids were detected in a photodiode array detector (Shimadzu model SPD-M10AVP) which collected spectra between 200 and 500 nm. Calibration curves for retinoids were obtained with standard, pure solutions of RAs. The detection limit for ROL and RAs was 2 pg [22,23]. Characteristic ultraviolet spectra and retention times for retinoids were identified, and peak areas were measured at λ_max-330 _in a Shimadzu SZ-228 data system.

### Plasma viral load

Plasma viral load was quantified by the Amplicor HIV-1 Monitor Test, version 1.5 (Roche Diagnostic Systems, Inc., Branchburg, NJ) with the lower limit of detection of 50 (1.7 log_10_) HIV-1 RNA copies/mL.

### Lymphocyte phenotyping

Lymphocyte phenotyping was performed in a FACS Calibur flow cytometry system (Becton Dickinson, San Jose, CA) after staining with the following monoclonal antibodies: anti-human CD3-FITC, CD8-PE, CD4-APC, CD45-PerCP, anti-human CD3-FITC, CD16-PE, CD56-PE, CD19-APC, CD45-PerCP and anti-human CD8-PE, CD38-FITC, CD45-PerCP (BD Biosciences, Mississauga, ON, Canada). CD38 density expression on CD8^+ ^was reported as median relative fluorescence index.

### Metabolic assessments

Fasting serum cholesterol, triglycerides and C-peptide were measured by standard techniques.

### Statistical analysis

We used only nonparametric statistical tests because we could not assume a normal distribution of data and the sample sizes were not large enough to rely on tests for normality. Correlations between retinoid concentrations and immuno-virological (i.e. CD4^+ ^cell count and percentages, CD8^+^38^+ ^fluorescence index and VL) and metabolic data (cholesterol, triglycerides and, in HIV-infected persons, C-peptide), were analyzed by Spearman's rank test. Differences between groups were determined by the Mann-Whitney rank test when the 2 HIV-infected groups were compared, and by the Kruskal-Wallis test followed (if P < 0.05) by Dunnett's multiple post-test comparisons when the 3 groups were compared. Changes over time in G1 were assessed by using Friedman's non-parametric test, followed (if P < 0.05) by Dunnett's post test. Statistical significance was accepted when P < 0.05. Statistical analyses were performed and graphic presentations created, using GraphPad Prism version 4.02 for Windows (GraphPad Software, Inc., San Diego, CA).

## Results

Baseline descriptors are presented in Table 1.

**Table 1:** Baseline descriptors

Descriptor	Group 1 N = 10	Group 2 N = 12	Group 3 N = 28	P value*
**Age (years)**				**P = 0.06**
Median (range)	41.5 (36-51)	48.4 (35.4-53^.^5)	36.5(21.0-63.0)	
Mean (SEM)	42.7 (1.8)	47.5 (1.4)	38.6 (2.4)	
95% CI	38.7, 46.7	44.5, 50.6	33.6, 43.5	
Triglycerides (mmol/L)**				**P < 0.01**:G1vs.G3 **P < 0.01**:G2 vs.G3
Median (range)	2.3 (1.1-4.6)	2.0 (0.7-5.4)	0.8 (0.4-3.0)	
Mean (SEM)	2.4 (0.3)	2.5 (0.4)	1.1 (0.1)	
95% CI	1.7, 3.1	1.5, 3.4	0.8, 1.3	
Cholesterol (mmol/L)**				**P > 0.05**
Median (range)	5.5 (3.9-7.7)	4.0 (1.5-6.4)	4.8 (3.2-7.0)	
Mean (SEM)	5.3 (0.4)	4.0 (0.4)	4.8 (0.2)	
95% CI	4.5, 6.2	3.2, 4.7	4.5, 5.2	

* P values using Kruskal-Wallis test followed by Dunnett's test for multiple comparisons

** For conversion from SI units to metric: for triglycerides mmol/L × 88.57 and for cholesterol mmol/L × 38.67.

Group 1 included all 10 (all male) participants in the CTN-140 study. Their cART consisted of D4T, 3TC, ddI plus indinavir (4 participants), ritonavir/saquinavir (4 patients), nelfinavir (1 patient), efavirenz (1 patient). Median (range) time duration on cART and off cART periods when retinoids were quantified was: ON 1 = 37.5 (22-48) months; OFF 1 = 16 (12-149) weeks; ON 2 = 21 (14-33) weeks; and OFF 2 = 23 (11-45) weeks. The duration of ON 2 was significantly shorter than that of ON 1 because the criteria to interrupt cART in CTN 140 study were an undetectable VL and CD4^+ ^T-cell count higher than 200 cells/mL, twice at 1 month interval. This allowed us to assess the effects of short-term optimal cART, in addition to the effects of long-term optimal cART during ON1.

Group 2 included 12 (10 male) HIV-infected adults with suboptimal cART (repeated detectable VL) consecutively seen at the same outpatient clinic: one who just stopped a suboptimal cART and 11 receiving 2 nucleoside reverse transcriptase inhibitors (NRTIs) plus nelfinavir (1 patient), a third NRTI (1 patient) or ritonavir (r)-boosted PIs (9 cases). The baseline descriptors of the 2 groups of HIV-infected adults are presented in Table 2. The 2 groups were similar in age, duration of HIV infection, duration of cART and body mass index (BMI). However, they significantly differed in terms of CD4^+ ^T-cell count and percentage, CD8^+^38^+ ^fluorescence index and VL, illustrating the differences between optimal versus suboptimal cART.

**Table 2:** Baseline descriptors for the 2 groups of HIV-infected participants

Descriptor	Group 1 (N = 10)	Group 2 (N = 12)	P value*
**HIV duration (years)**			**P = 0.2**
Median (range)	10.7 (1.5-13.0)	12.0 (2.0 - 18.0)	
Mean (SEM)	9.9 (1.2)	12.3 (1.4)	
95% CI	7.0, 12.7	9.2, 15.4	
**cART duration (years)**			**P = 0.1**
Median (range)	4.6 (1.4 - 11.0)	10.5 (5.0 - 15.0)	
Mean (SEM)	5.6 (1.2)	8.4 (1.4)	
95% CI	2.8, 8.3	5.2, 11.5	
**CD4^+ ^count (cells/μL)**			**P = 0.003**
Median (range)	375 (220-1050)	55.0 (10.0-520)	
Mean (SEM)	492 (95.0)	135 (54.0)	
95% CI	277, 707	16.0, 254	
**CD4^+ ^%**			**P = 0.002**
Median (range)	22.0 (15.0 - 43.0)	4.0 (1.0 - 26.0)	
Mean (SEM)	26.4 (3.3)	8.1 (2.6)	
95% CI	18.9, 33.9	2.3, 13.8	
**CD8^+^38^+ ^index**			**P = 0.001**
Median (range)	1.4 (0.9 - 2.5)	4.4 (1.5 - 12.6)	
Mean (SEM)	1.5 (0.2)	4.9 (0.9)	
95% CI	1.1, 1.9	2.9, 6.9	
**HIV viral load**			**P < 0.001**
(log_10 _RNA copies/mL)			
Median (range)	1.7 (1.7 - 1.7)	4.6 (1.7 - 6.2)	
Mean (SEM)	1.7 (0)	4.1 (0.4)	
95% CI	1.7, 1.7	3.2 (5.0)	
**Body mass index (kg/m^2 ^)**			**P = 0.7**
Median (range)	21^.^4 (19.1-30.1)	23.0 (16.6 - 29.8)	
Mean (SEM)	23.0 (1.2)	23.0 (1.0)	
95% CI	20.0, 25.8	20.7, 25.2	

*By using Mann -Whitney test

Group 3 comprised 28 (10 males) healthy adult volunteers (4 of the authors, laboratory staff, health-care workers, pharmaceutical representatives). They were younger than HIV-infected persons and had significantly lower triglyceride levels than G1.

### Serum RAs

**Figure 1:**
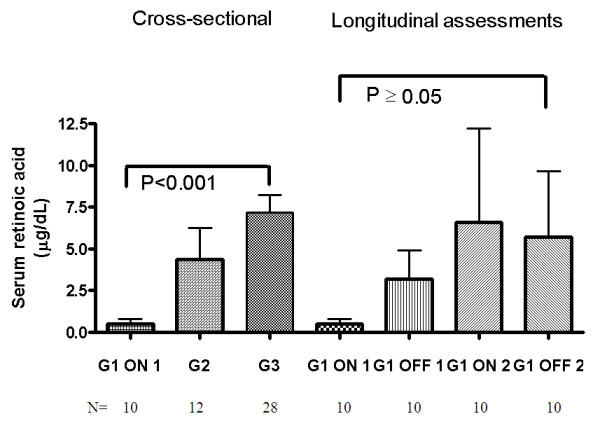
**Serum retinoic acid: cross-sectional and longitudinal assessments**. Bars represent means and SEM. **G1 **= patients enrolled in the CTN-140 Trial: ON 1 = during cART intensification (long-term effect); OFF 1 = during first cART interruption; ON 2 = after first cART resumption when viral load was again below the detection limit (short-term effect); OFF 2 = during second cART interruption. **G2 **= HIV-infected persons with suboptimal virologic control (repeated detectable VL); **G3 **= healthy volunteers. **P values **for cross-sectional assessments are from the Kruskal-Wallis test, followed by Dunnett's post hoc test for multiple comparisons. **P values **for longitudinal assessments in G1 are from Friedman's test, followed by Dunnett's post hoc test for multiple comparisons.

The serum RAs concentrations in the 3 groups are shown in Figure 1.

Serum RAs concentrations were statistically significantly lower in G1 while on long-term optimal and intensified cART (ON 1) in comparison to healthy adults who had the highest RAs values. Serum RAs were also markedly lower than in G2. At subsequent measurements (twice when off cART and once when re-initiated cART was fully suppressive) in G1 participants RAs levels did not changed significantly (P = 0.8), but a great interindividual variability was observed. However, the values in 75% percentile showed decreased levels during therapy and increased values while off treatment: ON1 = 1.3 μg/dL; OFF1 = 9.1 μg/dL;ON2 = 3.98 μg/dL and OFF2 = 7.96 μg/dL.

No correlation was found between serum RAs concentrations and VL, CD4^+ ^T-cell count or the CD8^+^38^+ ^fluorescence index in both groups of HIV-infected patients.

RAs did not correlate with fasting blood cholesterol or triglycerides level, which could be affected by cART or HIV infection. However, a significant correlation (r_s _= 0.8, P = 0.009) was found with C-peptide levels during re-initiated cART (ON 2) in G1.

### Serum ROL

**Figure 2:**
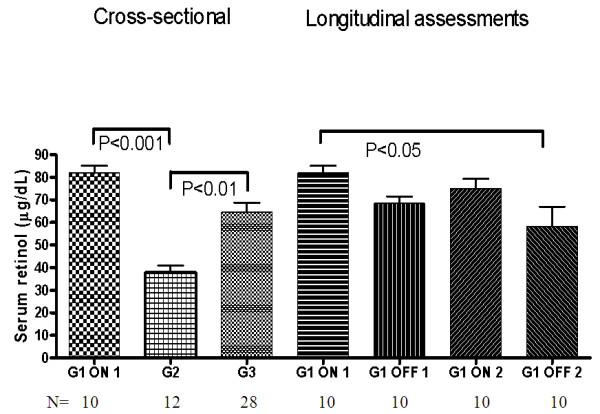
**Serum retinol: cross-sectional and longitudinal assessments**. Bars represent means and SEM. **G1 **= patients enrolled in the CTN-140 Trial: ON 1 = during cART intensification (long-term effect); OFF 1 = during first cART interruption; ON 2 = after first cART resumption when viral load was again below the detection limit (short-term effect); OFF 2 = during second cART interruption. **G2 **= HIV-infected persons with suboptimal virologic control (repeated detectable VL); **G3 **= healthy volunteers. **P values **for cross-sectional assessments are from the Kruskal-Wallis test, followed by Dunnett's post hoc test for multiple comparisons. **P values **for longitudinal assessments in G1 are from Friedman's test, followed by Dunnett's post hoc test for multiple comparisons.

Serum ROL concentrations are presented in Figure 2.

G1 participants had the highest ROL levels (82 ± 3 μg/dL) but not significantly higher from those in G3 that were within reported values for North Americans. ROL concentrations were significantly decreased in G2. In G1, ROL declined during cART interruptions when VL was detectable. Although ROL concentrations rose during cART resumption (ON 2) they did not reach initial values, and decreased significantly (P < 0.05) during the second cART interruption. In G1, while on cART, serum ROL correlated with triglycerides and cholesterol levels. No correlation was apparent between ROL and VL, CD4^+ ^T-cell count or CD8^+^38^+ ^fluorescence index in HIV-infected patients.

No correlation was found between serum ROL and concomitant RAs concentrations in the whole study population.

### ROL/RAs ratio

We assessed also this ratio since both parameters could be affected: ROL, mainly by HIV infection and RAs mainly by cART.

**Figure 3:**
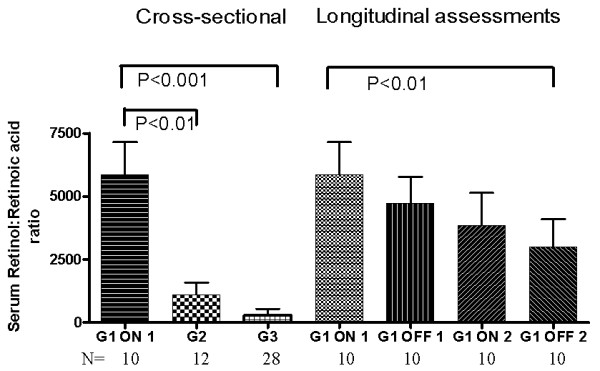
**Serum retinol: retinoic acid ratio: cross-sectional and longitudinal assessments**. Bars represent means and SEM. **G1 **= patients enrolled in the CTN-140 Trial: ON 1 = during cART intensification (long-term effect); OFF 1 = during first cART interruption; ON 2 = after first cART resumption when viral load was again below the detection limit (short-term effect); OFF 2 = during second cART interruption. **G2 **= HIV-infected persons with suboptimal virologic control (repeated detectable VL); **G3 **= healthy volunteers. **P values **for cross-sectional assessments are from the Kruskal-Wallis test, followed by Dunnett's post hoc test for multiple comparisons. **P values **for longitudinal assessments in G1 are from Friedman's test, followed by Dunnett's post hoc test for multiple comparisons.

ROL/RAs ratios (Figure 3) were significantly higher in G1 than in the other 2 groups but decreased significantly during the second cART interruption (P < 0.01). No correlation was found between the ROL/RAs ratio and VL, CD4^+ ^T-cell count, or the CD8^+^38^+ ^fluorescence index while on or off cART.

ROL/RA ratio correlated significantly (r_s _= 0.76, P = 0.01) with fasting serum cholesterol in G1 during ON2 (short term optimal cART).

### Gender differences in RAs and ROL levels

Since our study participants were not matched by gender we analysed also data from adult males only:10 in each group. We found the same statistically significant differences, as we have seen with the entire study population. Serum ROL levels in G1 were the highest and statistically significantly greater than in G2 males (P < 0.001). Healthy males from G3 had statistically significantly elevated ROL levels than male patients from G2 (P < 0.01). We also noted the same statistically significant difference (P < 0.01) in RAs concentrations between G1 and the 10 men from G3. Moreover, there were no significant differences between serum RAs (P = 0.6) or ROL levels in males only versus the entire group of participants. No significant difference was found between healthy males and females for RAs (P = 0.5) or ROL (P = 0.2).

## Discussion

This work provides evidence that serum retinoid concentrations are affected in HIV-infected adults and that both cART and HIV infection are contributing factors. An optimal cART and, to a lesser degree, a suboptimal cART, drastically diminished serum RAs concentrations in HIV-infected adults in comparison to healthy volunteers. This effect was more pronounced and statistically significant in patients with intensified and prolonged optimal cART. Longitudinal assessments in these patients while on or off cART did not show significant changes. This could be due to the low number of participants, great interindividual variability and mostly to the different duration of ON1 versus ON2 and OFF1 versus OFF2. However, if we look at the 75% percentile we see the same "pattern": RA levels increase during cART interruptions and diminish when cART is re-initiated. Decreased serum RA concentrations during cART is probably the result of altered intracellular retinoid metabolism by cART. We previously demonstrated that some antiretrovirals increase *in vitro *activity of RALDH1 and, consequently, RAs synthesis [17]. Moreover, one protease inhibitor, indinavir, also augmented RALDH1 mRNA expression [17]. *In vivo*, such antiretrovirals might also affect intracellular RALDH1, and increase intracellular RAs concentrations especially in those tissues actively involved in retinoid metabolism, like adipose tissue, in which they penetrate, and accumulate [24,25]. However, not all PIs have the same effect since they enter and accumulate differently in different tissues and have different intracellular localizations [21,25,26]. Moreover, as it was recently reported, adipose tissue influences tissue distribution of carotenoids [26] and certainly of RAs [24]. Heightened RAs concentrations in different tissues [21] enhance the expression of various P450 CYP enzymes such as CYP 26A1, CYP 26B1 and CYP 26C1, resulting in increased RAs catabolism [10,27]. Of note, these CYP enzymes are different than those affected by PIs. Furthermore, elevated intracellular RAs concentrations have a negative feedback action and reduce their own synthesis by lowering RALDH1 expression [28]. Therefore, it is likely that the low serum RAs concentrations in patients with optimal cART could be due to increased RAs catabolism and feedback inhibition of their synthesis that followed increases in their intracellular concentration when cART was initiated. In order to document this assumption, concomitant measurements of serum and tissue RAs concentrations are necessary. This was not possible when this study was undertaken because our technique was not suited, at that time, for tissue samples processing. Altered retinoid metabolism could have multiple consequences by affecting RAs-dependent genes involved in metabolic, hormonal and immune processes [4,6,1,23] and may explain some reported HIV- and cART-related metabolic and hormonal abnormalities [23].

It was shown previously that retinoids can modulate HIV-1 long terminal repeat-directed expression and either augment or reduce HIV replication according to cell line and culture conditions [29,30]. It was also reported that all-*trans *RA may act as a reverse transcriptase inhibitor reducing the HIV-1 proviral DNA load [31]. In both our groups of HIV-infected persons, serum RAs concentrations were not correlated with viral load, or CD4^+ ^T-cell count or CD8^+ ^38^+ ^fluorescence index. However, such correlations might exist between intracellular RAs concentrations and viro- immunological results. It is noteworthy that in G1 patients, RAs were significantly correlated with fasting serum C-peptide when their cART was reinitiated after the first cART interruption. This is consistent with the reported correlation between RAs and hemoglobin A1c in diabetes mellitus patients [32].

We also showed that ROL concentrations were highest during intensified, optimal cART, and they decreased during cART interruptions when HIV load rebounded, and then increased slightly when cART was resumed and VL was again undetectable. We observed also that HIV-infected adults with suboptimal cART have significantly lower ROL concentrations than patients with optimal cART and healthy adults. These data confirm our previous results [17] and are in keeping with other reports [17-19]. These observations clearly underline the negative effect of HIV infection on serum ROL and the beneficial effects of optimal cART.

Decreased serum ROL concentrations have been noted in HIV-infected individuals since the beginning of the AIDS epidemic, and they have been correlated with HIV-related morbidity and mortality as well as mother-to-child HIV transmission [19,33,34]. In fact, it is well known that plasma ROL decreases during inflammation and infection, including HIV [35]. In one study, patients with raised C-reactive protein levels had ROL concentrations lower by 25% [35]. It is suggested, therefore, to adjust serum ROL measurements with the concomitant values of inflammatory markers, such as C-reactive protein, in order not to overestimate vitamin A deficiency [35]. Although we routinely measure C-reactive protein levels in our HIV-infected patients, this was not included in the initial protocol. If we retrospectively adjust serum ROL levels during uncontrolled HIV infection by increasing the measured values by 25% for C-reactive protein elevation as suggested [35] we still have significant differences between G1 and G2.

Vitamin A supplementation has been shown, in some reports, to be beneficial in children, women and HIV-infected people in developing countries [29,33,34]. However, other reports showed that vitamin A and β-carotene supplementation in lactating women increases HIV load in breast milk [36]. Furthermore, vitamin supplementation, including vitamin A and β-carotene, increases the risk of subclinical mastitis in HIV-infected women [37]. A systematic review of randomized trials did not support vitamin A supplementation of HIV-infected pregnant and lactating women, despite improvement in birth weight [38]. A Cochrane review also found that currently available evidence does not support the use of Vitamin A supplementation of HIV-infected pregnant women to reduce mother-to-child transmission of HIV [39]. The best way to normalize or increase ROL levels is to optimally treat HIV infection as our data clearly showed.

The elevated ROL concentrations detected during optimal cART are certainly the result of appropriate control of HIV infection, and, probably, of improved epithelial integrity and increased intestinal absorption [10,40,41]. Decreased ROL utilization is also possible, due to decreased RAs synthesis. The ROL concentrations correlated with serum triglycerides and cholesterol in G1 while on cART, suggesting that both ROL elevation and these metabolic abnormalities are, partly, related to cART. Moreover, HIV-infected patients had significantly higher baseline triglycerides levels in comparison with healthy volunteers. Elevation of serum triglyceride concentrations is a known adverse effect of some antiretrovirals [15] and is also reported in patients treated with RA [12]. This finding indirectly suggest that cART-related hypertriglyceridemia might be secondary to increased intracellular RA levels.

Finally, we observed that ROL/RAs ratios are significantly elevated during cART, especially during its intensification, as compared with healthy controls and people with suboptimally-controlled HIV infection. Both increased ROL and diminished RAs levels were responsible for such high ratios.

The limitations of this study should be also considered, mainly the reduced sample size and uneven gender distribution. We had only 10 participants in CTN 140 trial due to ethical requirements in 1999 when this clinical trial was designed, and only 12 consecutive patients with suboptimal cART when the cross-sectional assessments were performed. However, in spite of this small sample size, we demonstrated statistically significant differences between groups and intra subjects followed longitudinally. As to the gender, there are no clear data showing gender differences in retinoid metabolism [35]. We did not find significant differences between healthy males and health females. When we analysed separately the male persons only, in spite of the reduced sample size of 10 patients in each group, we obtained similar results as for the whole group of participants.

HIV-infected persons were receiving different cART regimens when the tests were performed and this might be another limitation. However, the study was not designed to assess the effects of different cART on serum retinoids but rather the effects of optimal versus suboptimal cART and of HIV alone during cART interruptions. Another limitation is the lack of a control group of naïve-to-treatment HIV-infected persons. This was not possible when this exploratory study was initiated because very few naïve patients were seen at our center and most of them were hospitalised for AIDS-related illnesses. Furthermore, the effects, if any, of therapeutic vaccination and hydroxyurea (used to diminish the lymphocyte activation) could not be totally excluded. However, the intra-subject approach with longitudinal assessments diminished this theoretical bias. Moreover, hydroxyurea has a short half-life and is was given as a single dose in the evening and the blood specimens for retinoid assessments were drawn more than12 hours after the dose. Finally, being an exploratory work we did not assessed concomitant intracellular retinoid levels.

Nevertheless, we demonstrate that serum retinoids are significantly altered in adults with chronic HIV infection and that the contributing factors could be both HIV infection and its treatment. Based on these data and previous *in vitro *work we may assume that some of the retinoid-like side-effects, including metabolic abnormalities or clinical events, seen in HIV-infected persons on cART are due, at least in part, to altered intracellular retinoid metabolism [23]. Moreover, other beneficial non-virologic effects of antiretrovirals might be related to the effects of cART on retinoid metabolism [23]. However, further studies assessing concomitant serum and intracellular retinoid levels during different cART regimens in larger, homogenous groups of HIV-infected persons are warranted.

## Competing interests

The authors declare that they have no competing interests.

## Authors' contributions

The authors' contributions were as follows-ML: elaboration of patient's consent forms and information for participants, healthy controls enrolment, study design, HPLC analysis, data collection and interpretation, figures and manuscript preparation; HB: HPLC technique adaptation for human samples, serum analysis, data interpretation and manuscript preparation; SD-serum analysis, data interpretation, manuscript preparation; DP: study design, provision of significant clinical advice and manuscript preparation; PVB: overall laboratory supervision, HPLC technique development, design of the study, data interpretation and manuscript preparation; ET: study design and coordination, patients enrolment and follow-up, statistical analysis and data interpretation, manuscript preparation.

All authors have read and approved the final manuscript and its revised version.
